# Supplementary material for: Flowering and Seed Production across the Lemnaceae
Source: Int J Mol Sci. 2021 Mar 8;22(5):2733. doi: 10.3390/ijms22052733 (PMC7962950; doi:10.3390/ijms22052733)
Supplement: Supplementary file 1 [file ijms-22-02733-s001.zip › Supplementary Figures.docx]

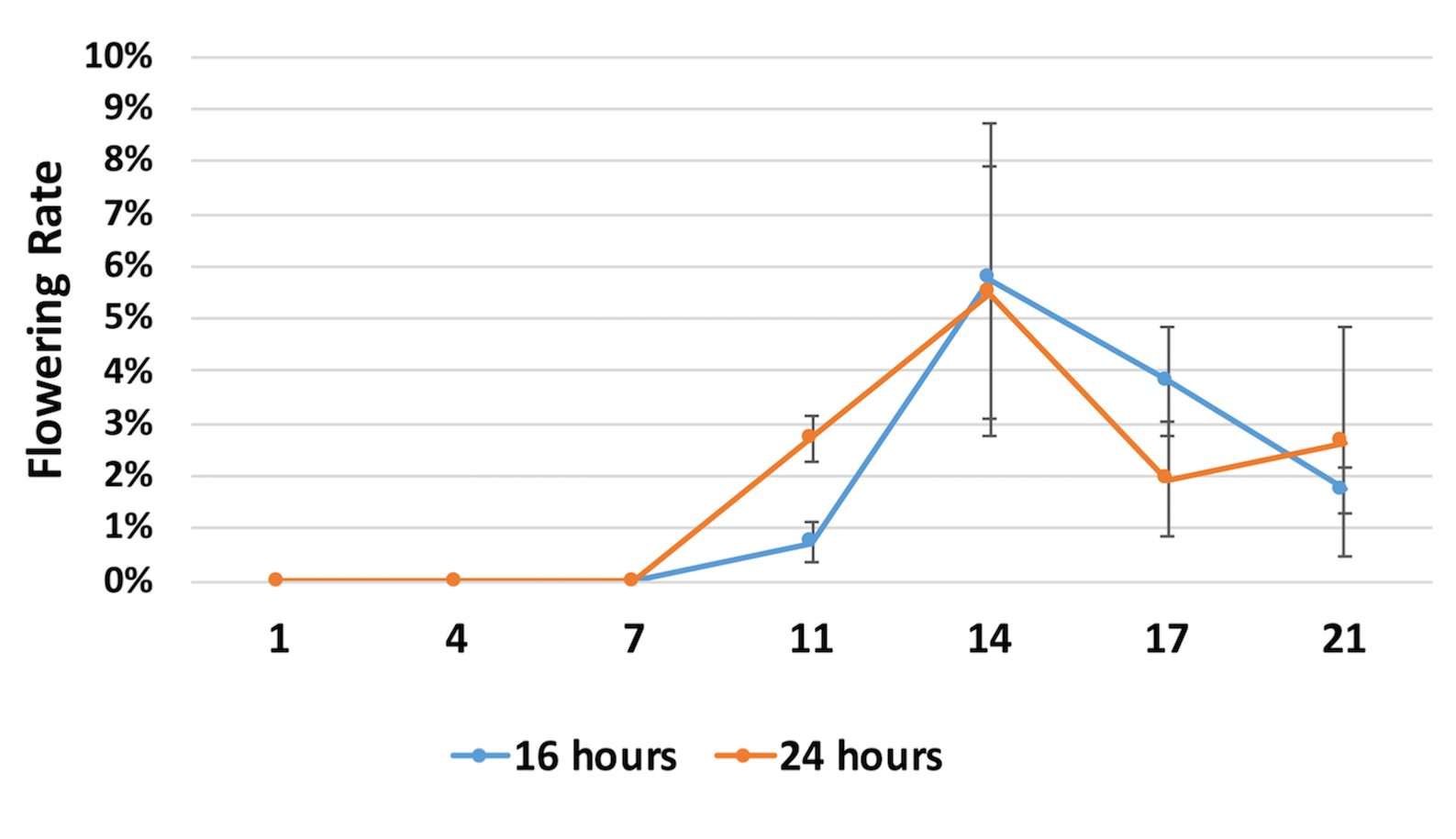


**Figure S1:**Flowering rates of *S. polyrhiza* cultures growing on Hg inoculated with fronds from a 4 week old culture were independent of photoperiod, although flowering occurred earlier in the culture cycle under CL conditions as compared to LD. Data are the average ± SEM (n = 3).


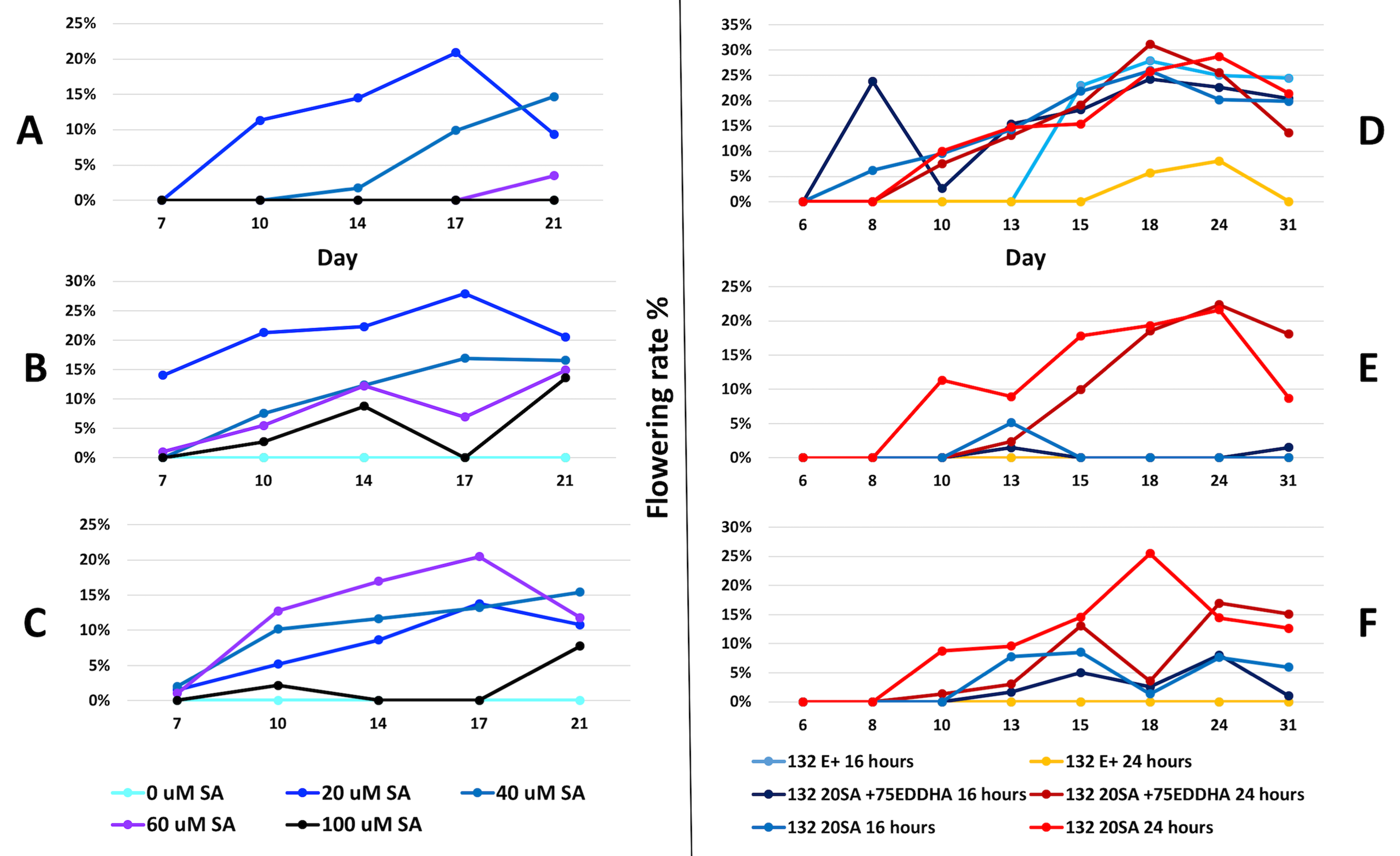


**Figure S2.** Screen for the effects of 0–100 µM SA and 75 µM EDDHA in E on flowering rate of *L. gibba* G3 and 2 strains of *L. minor* in LD or CL. Flowering rates of DWC114 (**A**), DWC131 (**B**), and DWC132 (**C**) in flasks of E with 0–100 µM SA under CL. (n = 1). Flowering rate in LD or CL with E media, E + 20µM SA, and E + 20 µM SA + 75µM EDDHA for strain DWC114 (**D**), DWC131 (**E**), and DWC132 (**F**) (n = 1).


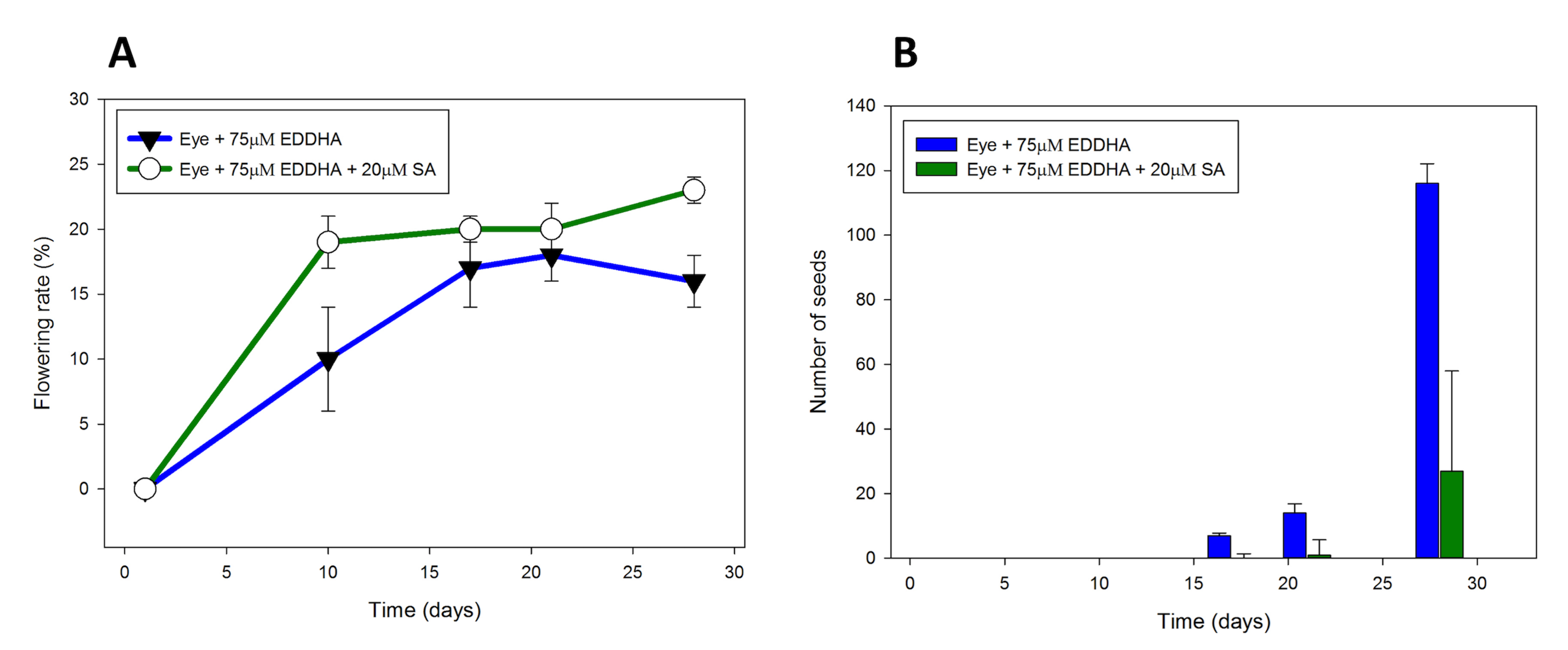


**Figure S3.** Flowering rate and seed production in strain DWC114 (**A**) Flowering rate in Eye media. Data are the average ± SEM (n = 3). * indicates a significant difference (*p* < 0.05), ** indicates *p* < 0.01, between the two conditions (n = 3) according to Student’s *t*-test. (**B**) Total number of seed produced, with the same statistical analysis.
