# Supplementary material for: Flowering and Seed Production across the Lemnaceae
Source: Int J Mol Sci. 2021 Mar 8;22(5):2733. doi: 10.3390/ijms22052733 (PMC7962950; doi:10.3390/ijms22052733)
Supplement: Supplementary file 1 [file ijms-22-02733-s001.zip › Lemnaceae Flowering Supplemental Document 1.doc]

# Supplemental Document 1

# E Medium Recipe (from C. Cleland)

# Stock Solutions for E Medium

Prepare the following stock solutions using glass distilled water or the best water you can get.

M**ajor Elements:	g/500 ml**

Ca (NO_3_)_2_ ·4H_2_O	29.50 g

KNO_3_	37.88 g

KH_2_PO_4_	17.00 g

*Need to add ~ 3 ml of 6N HCL for salts to dissolve.

Stir until salts are dissolved (approx. 2hr.)

# Trace Elements: mg/500 ml

## H_3_BO_3_ 143 mg

## ZnSO_4_ ·7H_2_O 11 mg

## Na_2_MoO_4_·2H_2_O 6 mg

## MnCL_2_·4H_2_O 181 mg

**CuSO_4_·5H_2_O	4 mg

(** Inhibits flowering. We omit it from E-Medium)

# Tartaric Acid: (autoclave or filter sterilize) mg/100 ml

Sodium –Potassium Tartrate or	564 mg

Tartaric Acid	300 mg

**Iron:**	**mg/100 ml**

## FeCL_3_·6H_2_O 108 mg

**EDTA:**	**mg/100ml**

EDTA (free acid)	900 mg

Need ~4 ml of 6N KOH to dissolve EDTA

**Magnesium:**	**g/500 ml**

## MgSO_4_·7H_2_O) 25 g

**To Prepare 1 Liter E medium**

To 10 g sucrose in a flask, add about 800 ml of glass distilled water.

Add:

20 ml of Major elements

10 ml of Trace elements

10 ml of Magnesium

5 ml of Iron

1 ml of Tartaric acid or Sodium Potassium Tartrate (Filter sterile)

1 ml of EDTA

Adjust pH to 4.6 with KOH

Bring volume up to 1 L with glass distilled water

Autoclave

NOTES :

- Eye Medium = E plus 600 mg/liter Bactotryptone

100 mg/liter Yeast Extract
